# Comparative Evaluation of the Removal of Gutta Percha From the Root Canal Using Various Retreatment File Systems With and Without Magnification: An In Vitro Study

**DOI:** 10.7759/cureus.62128

**Published:** 2024-06-11

**Authors:** Nishitha Arun, Pradeep Solete, Ganesh Jeevanandan, Delphine P Antony, Sruthi Sairaman, Swathi S

**Affiliations:** 1 Department of Conservative Dentistry and Endodontics, Saveetha Dental College and Hospitals, Saveetha Institute of Medical and Technical Sciences, Saveetha University, Chennai, IND; 2 Department of Pediatric and Preventive Dentistry, Saveetha Dental College and Hospitals, Saveetha Institute of Medical and Technical Sciences, Saveetha University, Chennai, IND

**Keywords:** solite rs3, retreatment, remnants, protaper, neo endo, gutta percha

## Abstract

Aim and objective: The main goal was to compare the efficacy of gutta percha (GP) removal from the root canal using the Neo Endo Retreatment file system, Solite RS3, and ProTaper Universal Retreatment (PTUR) files with and without magnification under a direct operative microscope using stereomicroscopic evaluation.

Materials and methods:Sixty single-rooted teeth were randomly assigned to one of three groups after obturation till F2 mastercone with resin sealer: Group 1 (n=20): Neo Endo Retreatment Files, Group 2 (n=20): Solite RS3, Group 3 (n=20): PTUR files. Each group was further separated into two subgroups: Subgroup 1: without magnification (no direct operative microscope) and Subgroup 2: with magnification under a direct operative microscope at 12× magnification. After retreatment, the roots were grooved buccolingually and split into two halves using a diamond disc with the help of a chisel. The samples were examined under a stereomicroscope. Images were captured in a digital camera and analyzed using image analyzing software Image Pro v10 (Media Cybernetics).

Results: The Neo Endo retreatment file system had a significantly greater percentage of remaining obturating material than the Solite RS3 Retreatment and PTUR file systems (p<0.05) in both groups with and without magnification. In the group without magnification, Solite RS3 showed a significant difference compared to ProTaper (p<0.05). In the group with magnification, there was no significant difference between the ProTaper Universal retreatment file system and Solite RS3 (p=0.589). Retreatment performed without magnification had more remnant GP when compared to the retreatment procedure performed under magnification of the direct operative microscope.

Conclusion:Under stereomicroscopic evaluation, the remnant GP was higher in the Neo Endo File System both with and without magnification than in the Solite Retreatment and PTUR file systems. ProTaper showed moderate significance in removing the obturation than Solite RS3 in the magnification group. The Solite RS3 file system performed as efficiently as the PTUR file system.

## Introduction

The primary objective of root canal therapy (RCT) is to uphold sterility within the root canal system through thorough disinfection, ultimately leading to the eradication of bacteria and its associated by-products. RCT is a common technique that is meticulously designed to treat infections that appear within the root canal system [1,2]. It entails the thorough removal of infected and inflammatory tissue by a method known as chemo-mechanical debridement, which uses multiple file systems to shape and clean the root canal area [2,3].

The establishment of a hermetic seal during obturation is also a crucial aspect of this treatment. The inability to attain this objective results in the recurring manifestation of clinical indications and mandates the requirement of endodontic retreatment [4]. Despite the good success rate of primary RCT ranging from 68% to 85%, the inadequacy of the initial RCT necessitates the requirement for retreatment [5]. The outcome of RCT is heavily reliant on the thorough elimination of necrotic and infected remnants and effective disinfection of the root canal system, culminating in a tightly sealed canal space. Failure to adequately fill these requisites can result in unsuccessful outcomes in RCT.

The presence of unlocated canals during endodontic treatment may lead to the persistence of irritants such as tissue and bacteria within the tooth structure [5-9]. A multitude of causative factors can be attributed to the failure of RCT, including iatrogenic mishaps, inadequate chemo-mechanical preparation, as well as issues with obturation and coronal seal. The existence of lingering microorganisms within the instrumented regions of the root canal may prompt the discharge of noxious byproducts that infiltrate the peri-radicular space by way of microleakage. This has the potential to instigate secondary infection [10]. The aforementioned necessitates a requirement for interventions, either non-invasive or invasive in nature. The most preferred option is non-surgical retreatment (NSRT) since it is deemed to be a more judicious and conservative approach compared to surgical retreatment.

Multiple variables exert an impact on the formulation of treatment plans in these circumstances. NSRT is frequently utilized in clinical practice, exhibiting a notable success rate ranging between 74% and 98%. The NSRT is commonly favored due to its practicality and capacity for conserving tissue as opposed to the more invasive surgical retreatment [11]. The fundamental goal of the NSRT procedure is to gain access to the apical foramina. This can be accomplished through a process of retrieving all materials, including gutta percha (GP) and sealer, utilized during the obturation procedure [12]. The removal of GP from the root canals can be achieved through mechanical, thermal, or chemical means. The thermal method is regarded as the most secure approach; however, its independent use is deemed insufficient.

The implementation of chemical techniques utilizing xylene or chloroform has been demonstrated to potentially pose a hazard in terms of toxicity to the periapical tissues. The mechanical technique has demonstrated effectiveness, but it can cause canal transportation and ledge formation, as reported in prior studies [7]. The application of ultrasonics in the effective removal of GP can also be employed [13]. At the outset, the employment of hand files, gates glidden drills, and Peeso reamers was commonly practiced for the retrieval of GP; however, these procedures have subsequently been supplanted by the utilization of nickel-titanium (NiTi) rotary instruments.

The use of conventional files to remove a well-condensed obturating substance has historically been an unpleasant and arduous technique for the operator, potentially leading to endodontic mishaps. Ni-Ti files' excellent flexibility allows for reasonably centered canal preparation with less canal carriage and a reduced frequency of canal defects. Additionally, the improved taper preparation enables appropriate irrigation. When used in a crown-down approach, these files provide a more efficient cutting action and a consistent reaming action. Despite their increased flexibility, Ni-Ti files have difficulty retrieving obturating materials from canal walls completely [14]. Clinicians are worried not only about the capacity of endodontic files and instruments to remove filling material properly but also about the level of safety given throughout the removal procedure.

In recent times, several retreatment file designs have been introduced, which are characterized by their cross-sectional shape, tip configuration, mode of operation (i.e. continuous or reciprocating), and utilization of diverse heat treatment methodologies such as CM-wire, M-wire, and blue technology [6-8]. The present study aimed to determine the efficacy of three different retreatment file systems in the removal of GP from the root canal space when performed with and without magnification under a direct operative microscope.

## Materials and methods

Ethical statement

This in vitro study received approval from the Scientific Review Board (SRB) of Saveetha Dental College and Hospitals with Reference no SRB/SDC/ENDO-2004/23/107.

Sample size calculation

The sample size for the present investigation was determined based on a prior study that evaluated canal transportation between two retreatment file systems using cone beam computerized tomography. The assessment determined that a sample size of 60 is necessary to achieve a power of 95% (1-β = 0.95, α = 0.05) [15].

Sample preparation

One hundred freshly extracted single-rooted mandibular premolar teeth with one root canal were collected and analyzed. Excluded were teeth with root canal obliteration and excessive curvature. Sixty teeth with single roots were chosen for the study based on inclusion criteria. To make the teeth uniform in length, teeth were decoronated from the apex at 16 mm. The working length was set at 1 mm short of the apex. ProTaper Gold, NiTi heat-treated rotary system was used for biomechanical preparation up to F2. Irrigation was carried out using 3% NaOCl, rinsed with saline between each file, and 17% EDTA was used as the final root canal rinse. The canal was dried with paper points before being obturated with F2 GP as a matched taper cone with resin-based sealer (AH plus sealer, Dentsply Sirona, Baden, Switzerland). Cavit was used for entrance filling. The teeth were stored in 100% humidity and 37 degrees Celsius for seven days to ensure adequate setting of the resin sealer. 

Retreatment procedure

The teeth were randomly assigned to one of three groups.

Group 1 (n=20): Neo Endo Retreatment Files (Neo Endo, London, UK)

Group 2 (n=20): Solite RS3 (Solite Dental, Chennai, India)

Group 3 (n=20): ProTaper Universal Retreatment (PTUR) files (Dentsply Maillefer, Ballaigues, Switzerland)

These were further separated into two groups:

Subgroup 1: without magnification (no direct operative microscope)

Subgroup 2: with magnification under direct operative microscope at 12× magnification (CARL ZEISS OPMI pico dental surgical microscope; Carl Zeiss, Germany).

During retreatment, no GP solvents were used, and throughout the entire retreatment procedure, 3% sodium hypochlorite and 17% EDTA were used as irrigants to remove the smear layer and debris.

The removal of GP along with the sealer plays an important role in retreatment; in the present study, the retreatment procedure was considered completed when no more GP shaving was present during the procedure; to ensure the removal of remanent debris and sealer from the root canal walls, 17% EDTA was used as an irrigant to remove the smear layer. All the procedures were done by a single operator for standardization purposes.

Retreatment file system

Group 1 (Neo Endo Retreatment file system): It consists of N1, N2, and N3 files that were employed sequentially. Light apical pressure was applied utilizing the crown down method at 350 revolutions per minute.

Group 2 (Solite RS3 Endodontic Retreatment file): It consists of RS1, RS2, and RS3 files that were used sequentially in the brushing motion and crown down method at 350 revolutions per minute.

Group 3 (PTUR file): It consists of D1, D2, and D3 files that were utilized sequentially in the brushing motion and crown down method at 500 revolutions per minute.

The roots were grooved buccolingually and split into two halves using a diamond disc with the help of a chisel. The samples were examined under a stereomicroscope. Stereomicroscopes offer two primary benefits: the ability to analyze opaque specimens and the provision of a three-dimensional perspective of the sample. Images were captured in a digital camera and analyzed using image analyzing software Image Pro v10 (Media Cybernetics, Rockville, MD). No differentiation was done between GP and sealer. While measuring the remanent obturating material, both halves of the root were considered. The percentage of remaining obturating material was determined by dividing the area of remnants of obturation material by the area of the entire root canal space multiplied by 100 [16]. The percentage of remaining GP in the root canals was compared between the three file systems and with and without magnification. The retreatment file system with the highest percentage of obturating material was deemed inefficient. This study focused on the overall efficiency of the retreatment file systems to retrieve the previous obturating material from the canal to correlate with the clinical use; hence, the entire root canal space was considered for analysis.

Statistical analysis

The data sheet was filled up with the percentage of remaining obturation material with and without magnification. This data was then transferred to IBM SPSS Statistics 23 (IBM Corp, Armonk, NY) for statistical analysis. Using the exported data, one-way analysis of variance (ANOVA) and Tukey's post hoc test for multiple comparisons were used to analyze the significance of variance across variables. The significance level was set at p <0.05.

## Results

**Figure 1:**
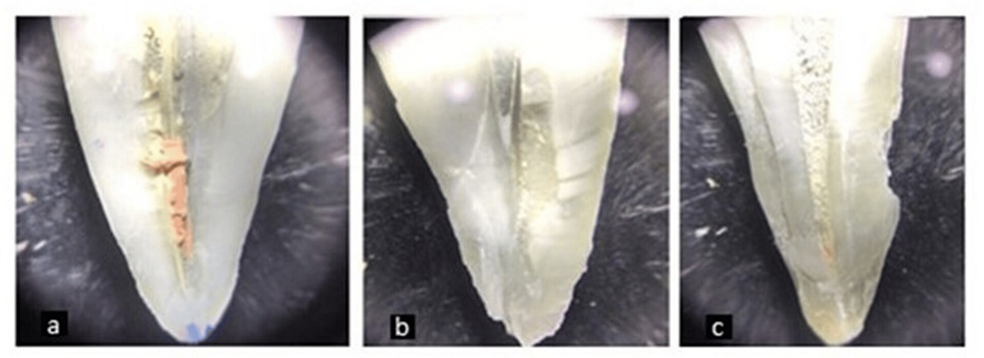
Remnant gutta percha viewed under stereomicroscope without magnification (a) Neo Endo file system; (b) Solite RS 3 Retreatment file system; (c) ProTaper Retreatment system.

In groups without magnification, the obtained mean percentage (%) of remnant GP and sealer was found to be as follows: Neo Endo group (31.92±3.52), Solite RS3 Retreatment file group (10.34±0.89), and PTUR (18.73±1.99). One-way ANOVA and post hoc Tukey showed the least remnants in Solite RS3 which is statistically significant compared to Neo Endo (p=0.005) and ProTaper (p=0.05). Between the ProTaper and Neo Endo groups, ProTaper showed a significant difference (p=0.038) (Table 1, Figure 1).

**Table 1:** Shows the percentage of remnant gutta percha under the stereomicroscope when the retreatment is not done under magnification aids Different capital alphabets in superscript represent the statistical difference between the three groups. Solite RS3 showed a statistically significant difference compared to the other two groups (p<0.05).

Groups	Mean±SD
Neo Endo	31.92±3.52^C^
Solite RS3	10.34±0.89^A^
ProTaper	18.73±1.99^B^

**Figure 2:**
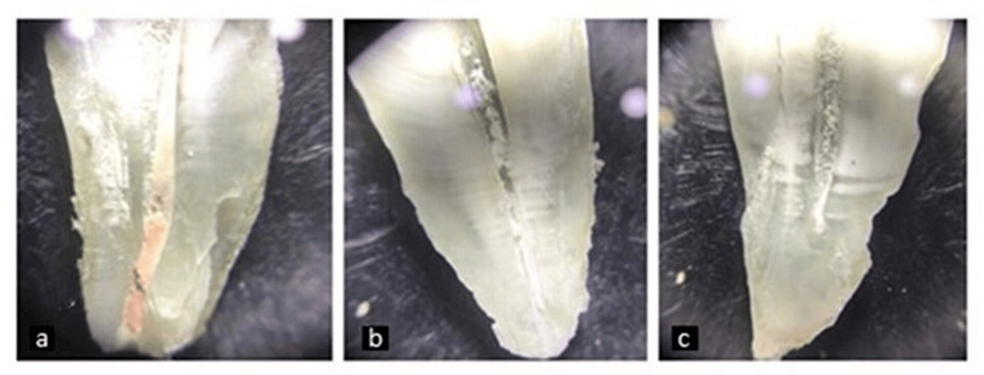
Remnant gutta percha viewed under stereomicroscope under magnification (direct operative microscope at 12× magnification) (a) Neo Endo file system; (b) Solite RS3 Retreatment file system; (c) ProTaper Retreatment system.

In groups with magnification, the obtained mean percentage (%) of remanent GP was found to be as follows: Neo Endo group (7.77±0.69), Solite RS3 Retreatment file group (3.48±0.80), and PTUR (3.81±0.67). One-way ANOVA and post hoc Tukey showed the least remnants in both Solite RS3 and ProTaper, which are statistically significant compared to Neo Endo (p=0.000), and no significant difference was seen between ProTaper and Solite RS3 groups (p=0.589) (Table 2, Figure 2).

**Table 2:** Shows the percentage of remnant gutta percha at 12.5× magnification under the stereomicroscope when the retreatment is done under the direct operative microscope at 12× magnification Different capital alphabets in superscript represent the statistical difference between the three groups. Solite RS3 and ProTaper showed a statistically significant difference compared to Neo Endo (p<0.05). No significant difference was seen between Solite RS3 and ProTaper (p>0.05).

Groups	Mean±SD
Neo Endo	7.77±0.69^B^
Solite RS3	3.48±0.80^A^
ProTaper	3.81±0.67^A^

**Figure 3:**
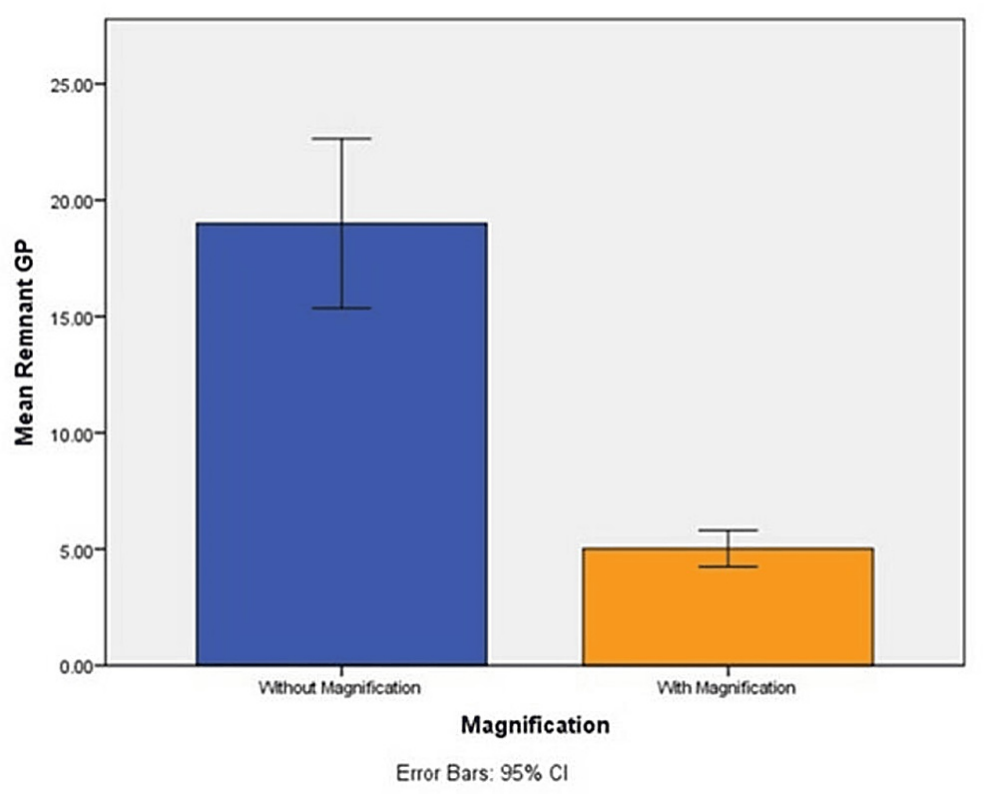
Remnant GP present was significantly higher in root canal without magnification when compared to that with magnification under direct operative microscope at 12× magnification (p<0.05) GP, gutta percha.

There was a significantly higher amount of remaining GP in groups without magnification compared to groups when retreatment was done under magnification using a direct operative microscope (p<0.05) (Figure 3).

## Discussion

The success rates of endodontic therapy are significantly dependent on the quality of root canal shape, cleaning, and microbial elimination [17]. Root canal filling porosity is closely related to poor endodontic treatment results [18]. The gaps inside the bulk of the materials might have a negative impact on their physical qualities and generate suitable circumstances for microleakage [19]. GP is the most often used root canal filler material in combination with a range of sealers. Because of its lack of adhesive properties, failure to reinforce the tooth after obturation, lack of stiffness, shrinkage on cooling, and hydrophobic nature, it often results in porosities in the root canal obturation [20].

Endodontic treatment failure can be due to inadequacies that occur during different phases of RCT as well as iatrogenic events [5,12]. Before implementing any therapeutic intervention, it is crucial to carefully consider all interdisciplinary therapeutic alternatives, considering their temporal, financial, prognostic, and potential impact on patient satisfaction [21]. NSER can be done in a single or numerous visits [22]. This method stands out for its practicality and greater patient compliance. When dealing with root canals with acute apical periodontitis and resistant endodontic lesions produced by chronic secondary infections, multiple-visit endodontic retreatment is preferred [23]. The multi-visit NSER technique seeks to improve disinfection by utilizing a comprehensive chemo-mechanical preparation routine, supporting robust recovery, and enhancing success rates in treating periradicular diseases [2]. The primary goals of NSRT involve the elimination of materials present within the canal space and the correction of any defect. In addition, non-invasive therapeutic measures confirm the presence of mechanical dysfunction, previously unidentified canal pathways, or fractures situated below the level of the root crest [24]. It is critical to remove all remanent obturating materials that prevent irrigating solutions and intracanal dressings from contacting the root canal walls during retreatment.

To remove GP from root canals, various methods have been used, including the use of K or H files with solvents such as eucalyptol, chloroform, xylene, or halothane, heated pluggers, and gates glidden drills, for coronal third material removal, followed by hand instrumentation or ultrasonic technique. Obturation material can also be removed from the root canals using flexible rotational tools in low-speed handpieces. The traditional use of hand instruments for removing obturation material took more time and was more arduous, whereas rotary methods removed the material from the root canal system faster. The heat created by friction on rotation plasticizes obturation materials, and the unique flute shape tends to suck the GP into the file flute, making obturation material removal more efficient [25].

The prevalent magnification instruments utilized in endodontics include loupes, surgical microscopes, and the more recently added endoscope. The use of such devices has become a widely established technique in both non-surgical and surgical endodontic therapy. In addition to enhancing the precision of endodontic procedures, these devices have the potential to enhance diagnostic capabilities by providing improved visualization and understanding of the area being treated [26]. The use of the dental operating microscope (DOM) helps in the understanding of complex structures, thereby permitting improved differentiation. The DOM provides improved visual perception with the use of magnified images, a brighter and more intense field of vision, and accurate maneuvering capabilities. The utilization of magnification during endodontic retreatment facilitates the removal of GP, enhancing accuracy and efficiency. Improved visualization and increased accuracy decrease the likelihood of problems and enhance the overall success rates of the procedure. Using magnification enables a thorough examination of the root canal, which allows for accurate detection of GP material present in the canal system. Improved identification of canal orifices is essential for efficiently accessing and removing GP [27]. In our current research, we found that the percentage of remaining GP was lower in groups where retreatment was performed with magnification compared to those where retreatment was done without magnification.

In the present study, when the Neo Endo Retreatment file system was used, there was a higher percentage of remaining GP in the root canal when compared to the other two file systems. This is in agreement with a study done by Sagare et al., which reported that Neo Endo files were less efficient than Wave-one file systems in removing GP from the root canal during retreatment [28]. The superior performance of PTUR files in the present study can be ascribed to their cross-section which is convex triangular that enhances their efficiency in cutting. When utilized in the absence of an irrigant or solvent, the engine-driven files generate heat within the canal, resulting in the plasticization of the GP resin. Consequently, the resin can be easily extracted from the canal [12]. Solite RS3 constitutes a file system composed of three components. Files are composed of an NiTi alloy and undergo a process of heat treatment to enhance their capacity to withstand fracture. This augmented durability permits the usage of such files in curved root canals, thereby facilitating endodontic procedures. Solite RS3 constitutes a file system composed of three components. Files are composed of an NiTi alloy and undergo a process of heat treatment to enhance their capacity to withstand fracture. Solite RS3 files undergo a process of heat treatment, resulting in enhanced flexibility. By being flexible, they are able to remove the obturating material from the root dentin without removing the root canal dentin. The Solite RS3 file features multiple cutting edges that make it easier to remove the obturating material. In addition, their remarkable flexibility serves as a safeguard against excessive dentin removal [15,29].

The limitation of the present study includes lesser sample size. Further in vitro and clinical studies comparing the efficacy of various retreatment file systems can provide clinical guidance to the usage of these files in endodontic retreatment.

## Conclusions

Under stereomicroscopic evaluation, the remnant GP was higher in the Neo Endo File System both with and without magnification than in the Solite RS3 and PTUR file systems. ProTaper was moderately significant in removing the remnant obturation than SoliteRS3 in the magnification group. Solite RS3 file system performed equally efficiently to the ProTaper Retreatment file system.

## References

[1] Estrela C, Holland R, Estrela CR, Alencar AH, Sousa-Neto MD, Pécora JD (2014). Characterization of successful root canal treatment. Braz Dent J.

[2] Gulabivala K, Ng YL (2023). Factors that affect the outcomes of root canal treatment and retreatment-A reframing of the principles. Int Endod J.

[3] Tomson PL, Simon SR (2016). Contemporary cleaning and shaping of the root canal system. Prim Dent J.

[4] Salehrabi R, Rotstein I (2004). Endodontic treatment outcomes in a large patient population in the USA: An epidemiological study. J Endod.

[5] Wong R (2004). Conventional endodontic failure and retreatment. Dent Clin North Am.

[6] Wahane KD, Kulkarni SS, Daokar S, Patil K, Patel K, Thorat T (2021). An assessment of the efficacy of a rotary and a reciprocating retreatment file system for removal of gutta-percha from root canals: An in vitro cone-beam computed tomography study. Endodontology.

[7] Azim AA, Wang HH, Tarrosh M, Azim KA, Piasecki L (2018). Comparison between single-file rotary systems: Part 1-Efficiency, effectiveness, and adverse effects in endodontic retreatment. J Endod.

[8] Rodrigues CT, Duarte MA, de Almeida MM, de Andrade FB, Bernardineli N (2016). Efficacy of CM-wire, M-wire, and nickel-titanium instruments for removing filling material from curved root canals: A micro-computed tomography study. J Endod.

[9] Schwendicke F, Göstemeyer G (2017). Single-visit or multiple-visit root canal treatment: Systematic review, meta-analysis and trial sequential analysis. BMJ Open.

[10] Hoen MM, Pink FE (2002). Contemporary endodontic retreatments: An analysis based on clinical treatment findings. J Endod.

[11] Saad AY, Al-Hadlaq SM, Al-Katheeri NH (2007). Efficacy of two rotary NiTi instruments in the removal of Gutta-Percha during root canal retreatment. J Endod.

[12] Indi S, Desai SR, Hambire A (2022). Comparison of the time required by six different retreatment techniques for retrieval of gutta-percha: An in vitro study. Eur J Gen Dent.

[13] Ward JR, Parashos P, Messer HH (2003). Evaluation of an ultrasonic technique to remove fractured rotary nickel-titanium endodontic instruments from root canals: An experimental study. J Endod.

[14] Agrawal P, Ramanna PK, Arora S, Sivarajan S, Jayan A, Sangeetha KM (2019). Evaluation of efficacy of different instrumentation for removal of gutta-percha and sealers in endodontic retreatment: An in vitro study. J Contemp Dent Pract.

[15] Valan AS, Solete P, Jeevanandan G, Priscilla Antony SD, Kavoor S (2023). Influence of heat-treated retreatment files on the canal transportation and centering ability during retreatment: An in vitro cone beam computed tomography study. J Int Oral Health.

[16] Muraleedhar AV, Satish SV, Patil AM, Kovvuru SK, Patil S (2021). Comparative evaluation of efficacy of three different rotary retreatment systems with manual instrumentation in removing gutta-Percha from root canals-An in-vitro study. J Evol Med Dent Sci.

[17] Kandemir Demirci G, Çalışkan MK (2016). A prospective randomized comparative study of cold lateral condensation versus core/gutta-percha in teeth with periapical lesions. J Endod.

[18] Moinzadeh AT, Zerbst W, Boutsioukis C, Shemesh H, Zaslansky P (2015). Porosity distribution in root canals filled with gutta percha and calcium silicate cement. Dent Mater.

[19] Marciano MA, Duarte MA, Camilleri J (2016). Calcium silicate-based sealers: Assessment of physicochemical properties, porosity and hydration. Dent Mater.

[20] Marfisi K, Mercade M, Plotino G, Duran-Sindreu F, Bueno R, Roig M (2010). Efficacy of three different rotary files to remove gutta-percha and Resilon from root canals. Int Endod J.

[21] Kvist T, Reit C (1999). Results of endodontic retreatment: A randomized clinical study comparing surgical and nonsurgical procedures. J Endod.

[22] Comparin D, Moreira EJ, Souza EM, De-Deus G, Arias A, Silva EJ (2017). Postoperative pain after endodontic retreatment using rotary or reciprocating instruments: A randomized clinical trial. J Endod.

[23] de Castro Kruly P, Alenezi HE, Manogue M, Devine DA, Dame-Teixeira N, Garcia FC, Do T (2022). Residual bacteriome after chemomechanical preparation of root canals in primary and secondary infections. J Endod.

[24] Lopes HP, Elias CN, Vedovello GA, Bueno CE, Mangelli M, Siqueira JF Jr (2011). Torsional resistance of retreatment instruments. J Endod.

[25] Prasad A, Nair R, Angelo JC, Mathai V, Vineet RV, Christopher S (2018). A comparative evaluation of retrievability of Guttapercha, Resilon and CPoints for retreatment, using two different rotary retrieval systems - An ex vivo study. Saudi Endod J.

[26] Del Fabbro M, Taschieri S (2010). Endodontic therapy using magnification devices: A systematic review. J Dent.

[27] Liu B, Zhou X, Yue L (2023). Experts consensus on the procedure of dental operative microscope in endodontics and operative dentistry. Int J Oral Sci.

[28] Sagare SV, Chandra P, Kaur T, Ganorkar O, Khade A, Mehta SD (2021). A comparative study of the efficacy of WaveOne and NeoEndo retreatment file system for the removal of gutta percha from the root canal. J Pharm Bioallied Sci.

[29] Arun N, Solete P, Jeevanandan G, Delphine Priscilla Antony S, Kavoor S, Sandeep AH (2023). Comparison of time required by three different retreatment file systems for retrieval of Gutta Percha-An in vitro study. J Popul Ther Clin Pharmacol.

